# The Expression of Key Ethylene and Anthocyanin Biosynthetic Genes of ‘Honeycrisp’ Apples Subjected to the Combined Use of Reflective Groundcovers and Aminoethoxyvinylglycine in the Mid-Atlantic US

**DOI:** 10.3390/plants13081141

**Published:** 2024-04-19

**Authors:** Md Shipon Miah, Macarena Farcuh

**Affiliations:** Department of Plant Science and Landscape Architecture, University of Maryland, College Park, MD 20742, USA; shipon@umd.edu

**Keywords:** *Malus domestica* Borkh, red skin color, anthocyanins, ethylene, reflective groundcovers, gene expression

## Abstract

The decreased profitability of important apple cultivars, such as ‘Honeycrisp’, results from the poor red skin coloration and high fruit drop in the mid-Atlantic US. Apple red skin coloration is determined by the anthocyanin concentration. Reflective groundcovers promote red skin coloration, whereas aminoethoxyvinylglycine (AVG) decreases the ethylene production and fruit drop, thus reducing the coloration. Although our previous study showed that combinations of these practices impact the fruit quality and color, research is lacking regarding their effects at the gene and metabolite levels. In this work, for two years, we compared the differences in the internal ethylene concentration (IEC), red skin coloration, fruit drop, transcript accumulation of key ethylene and anthocyanin biosynthetic pathway-related genes, and total anthocyanin concentration of ‘Honeycrisp’ apples. The fruit was treated with combinations of reflective groundcover (Extenday) and AVG (130 mg L^−1^) and was assessed throughout ripening. Extenday-only-treated fruit displayed the highest upregulation of ethylene and anthocyanin biosynthetic-related genes and of total anthocyanins, exceeding 50% blush, while boosting the IEC. In contrast, AVG significantly decreased the expression of key ethylene and anthocyanin biosynthetic-related genes and total anthocyanins, thus preventing apples from reaching 50% blush, while also decreasing the IEC and fruit drop. The combination of Extenday x AVG fine-tuned the transcript accumulation of ethylene and anthocyanin biosynthetic-related genes as well as the total anthocyanins, allowing the ‘Honeycrisp’ fruit to exceed 50% blush, while increasing the IEC moderately and reducing the fruit drop (as compared to Extenday-only and control), thus enhancing the fruit economic value.

## 1. Introduction

Red skin coloration in apple (Malus domestica Borkh) fruit is generally associated with a better marketability, consumer acceptability, and a higher profitability [1,2]. Additionally, at least a 50% blush is required for the commercialization of economically significant apple cultivars, including the top sales-producing cultivar ‘Honeycrisp’ [3,4,5].

The total anthocyanin concentration is a key component of apple red skin coloration [6]. Anthocyanins are classified as phenolic compounds, resulting from the phenylpropanoid pathway [7]. Besides their critical role in apple red skin coloration, anthocyanins also contribute to shielding the fruit from photooxidative damage, while also aiding in the removal of free radicals, consequently contributing to the reduction of cancers and coronary diseases, among others [8,9,10,11]. The presence of anthocyanins is usually higher in apple skin when compared to other tissues such as fruit flesh [12,13].

The anthocyanin biosynthesis pathway consists of numerous steps, starting with the precursor phenylalanine. Important enzymes that participate in this pathway comprise phenylalanine ammonia-lyase (PAL), chalcone synthase (CHS), chalcone isomerase (CHI), flavanone 3-hydroxylase (F3H), dihydroflavonol 4-reductase (DFR), leucoanthocyanidin dioxygenase (LDOX), and UDP glucose-flavonoid 3-O-glucosyltransferase (UFGT) [14]. Furthermore, regulatory genes, such as the transcription factor *MdMYB10*, have been reported to play key roles in red skin coloration by controlling the transcript accumulation of the structural genes involved in the anthocyanin biosynthetic pathway [1,15,16,17,18]. The expression of *MdMYB10* has been reported to be increased during the accumulation of anthocyanins in apple fruit [18].

The biosynthesis of anthocyanins primarily takes place during fruit ripening [19]. Other factors, such as light [20,21,22], as well as ethylene production [20,21,22,23,24,25,26,27], have been shown to drastically impact the anthocyanin concentration. Particularly regarding light, the accumulation of anthocyanins and the consequently increased red skin coloration has been shown to be affected by wavelength (stimulated via fruit exposure to ultraviolet radiation) and light intensity [21,28,29,30,31]. Light has been reported to induce the expression of MYB transcription factors, as well as of the structural anthocyanin biosynthetic genes [1,32,33,34,35,36,37].

Reflective groundcovers are a commercially available cultural practice that have been shown to increase the light that reaches the apple fruit surface via enhancing the light reflection (including ultraviolet radiation) from the orchard floor towards the tree canopy [3,4,28,38,39,40,41,42]. Consequently, the use of reflective groundcovers has been reported to promote red skin coloration in multiple apple cultivars grown under different environmental conditions [3,4,28,39,40,43,44,45,46], thus increasing crop value [3]. Although earlier studies have documented the effectiveness of reflective groundcovers on enhancing apple red skin coloration, research to evaluate the effect of this practice on the expression of anthocyanin biosynthetic pathway-related genes and the total anthocyanin concentration is scarce and has not been conducted under the environmental conditions of the US mid-Atlantic region.

The production of the hormone ethylene regulates anthocyanin biosynthesis [23,24,25,26,30,47]. Previous studies have shown that the initiation of anthocyanin biosynthesis and ethylene production concur [48]. However, ethylene production can also enhance fruit ripening, therefore directly impacting the fruit quality [4,49,50,51,52,53], and, at the same time, can promote the apple preharvest fruit drop [54]. The biosynthesis of ethylene has been significantly studied in fruit that present a climacteric behavior, such as apples, which display a rise in their rate of respiration and the internal ethylene concentration (IEC) as the ripening progresses [50,55,56]. Ethylene biosynthesis is driven by 1-aminocyclopropane-1-carboxylate synthase (ACS), which converts S-adenosyl-L-methionine (SAM) to 1-aminocyclopropane-1-carboxylate (ACC), and subsequently, by the action of ACC oxidase (ACO), drives the oxidation of ACC to ethylene [57,58]. Moreover, IEC has been shown to vary largely within apple cultivars. ‘Honeycrisp’, together with other cultivars such as ‘McIntosh’ and ‘Golden Delicious’, are high ethylene producers and have been classified as being highly prone to fruit drop, yielding losses than can reach up to 50% in some years, in contrast to ‘Fuji’ or ’Empire’, which are less susceptible to drop as the ethylene levels remain extremely low [59,60,61,62,63,64,65].

Aminoethoxyvinylglycine (AVG) is widely used in the apple industry as it has been shown to delay the apple-ripening rates and to reduce the preharvest fruit drop by significantly decreasing ethylene production via hampering the ACS activity [27,40,54,57,63]. Earlier studies have reported that the use of AVG has significantly delayed ripening and decreased fruit drop, but, at the same time, has negatively impacted red skin coloration in ‘McIntosh’, ‘Honeycrisp’, and ‘Gala’ cultivars, all grown under different environmental conditions [4,40,59,63,66,67,68,69,70]. Nevertheless, studies assessing the effect of AVG on ethylene and anthocyanin biosynthetic pathway-related genes and the total anthocyanin concentration, as well as fruit drop throughout ripening, are scarce for mid-Atlantic-grown ‘Honeycrisp’ apples. 

Research targeting the effects of the combined use of reflective groundcovers and AVG is limited, despite their potential capacity of concurrently solving the critical problems of poor red skin coloration and high fruit drop in ‘Honeycrisp’ apples. In our previous work, we demonstrated that the combination of both horticultural practices could impact the quality as well as the skin color of ‘Honeycrisp’ apples grown in the mid-Atlantic US [4]. However, to our knowledge, studies on the impacts of these treatment combinations on ‘Honeycrisp’ key ethylene and anthocyanin biosynthesis-related transcript accumulation, as well as on the total anthocyanin concentration and fruit drop under our environmental conditions, is lacking. Based on the above, the aim of the present work was two-fold: first, to characterize and compare the differences in the IEC, red skin coloration, fruit drop, transcript accumulation of key ethylene and anthocyanin biosynthetic pathway-related genes and transcription factors, as well as the total anthocyanin concentration of ‘Honeycrisp’ apples subjected to treatment combinations of reflective groundcover Extenday and AVG during ripening on the tree in the mid-Atlantic US; and, secondly, to use multivariate data analysis in order to detect important correlations amongst the assessed variables.

## 2. Results

### 2.1. Effects of Extenday and AVG Treatment Combinations on ‘Honeycrisp’ Internal Ethylene Concentration and Red Skin Coloration

**Figure 1:**
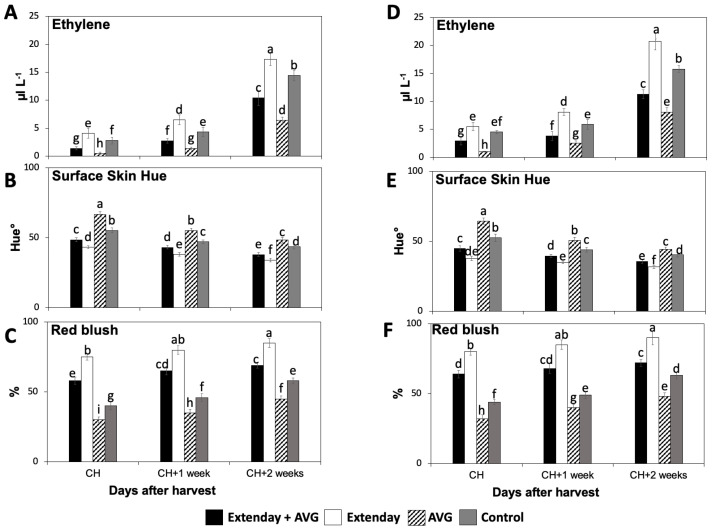
Effects of Extenday and AVG combinations on the internal ethylene concentration (IEC) and red skin coloration of ‘Honeycrisp’ fruit grown in Aspers, PA, in (**A**–**C**) 2021 and (**D**–**F**) 2022. Apples were evaluated at optimal commercial harvest (CH), 1 week after CH (CH + 1), and 2 weeks after CH (CH + 2). Values are means ± standard error. Different letters indicate significant differences (*p* ≤ 0.05) according to Tukey’s HSD test.

In this study, strong and consistent differences were observed in the internal ethylene concentration (IEC) and red skin coloration amongst the different Extenday and AVG treatment combinations for the two evaluated production seasons (Figure 1). 

For all assayed treatments, the IEC values were significantly augmented throughout the three assayed ripening stages (Figure 1A,D). In general, for each assessed ripening stage, Extenday-only (T2)-treated fruit always displayed the highest IEC, followed by control (T4) fruit, and next by the combined Extenday + AVG (T1) treatment, while AVG-only (T3)-treated fruit presented the statistically lowest IEC. This was consistent in both years, excluding CH in 2022, where T2 and T4 presented no differences (Figure 1D). 

Red skin coloration significantly increased throughout the three assayed ripening stages for all assessed treatments in both years, according to surface skin hue angle and skin blush percentage measurements (Figure 1B,C,E,F). In general, for each assessed ripening stage, the AVG-only (T3)-treated fruit presented the statistically highest values for the surface skin hue angle, followed by the control (T4) fruit, and next by Extenday + AVG (T1), while Extenday-only (T2) presented the lowest values (Figure 1B,E). Results for the skin blush percentage were consistent with the skin hue angle evaluations. For each assessed ripening stage, the Extenday-only (T2)-treated fruit always displayed the highest skin blush percentage (>75%), followed by the combined Extenday + AVG (T1) treatment (>50%), and next by the control (T4)-treated fruit (40–60%), while the AVG-only (T3)-treated fruit presented the statistically lowest skin blush percentage, only reaching the minimum required 50% blush at the last assessed ripening stage (CH + 2) (Figure 1C,F).

### 2.2. Effects of Extenday and AVG Treatment Combinations on ‘Honeycrisp’ Fruit Drop

The ‘Honeycrisp’ fruit drop significantly increased throughout the three assayed ripening stages for all treatments in both years (Table 1). However, in 2021, at all evaluation periods (CH, CH + 1, CH + 2), and in 2022 for CH + 1 and CH + 2, Extenday-only (T2)-treated fruit together with the control fruit (T4) presented similar levels of drop, which were 1.5- to 2-fold higher than the fruit where AVG was applied (T1 and T3). At the last evaluation period (CH + 2), the fruit that were treated with AVG (T1, T3) showed fruit drop values ~17% in 2021 and ~15% in 2022, which were significantly lower than those from fruit that had not received AVG (T2, T4), which showed fruit drop values ~30% in 2021 and ~25% in 2022.

### 2.3. Effects of Extenday and AVG Treatment Combinations on Key Ethylene and Anthocyanin Biosynthetic Pathway-Related Genes and Transcription Factors Associated with Their Regulation

#### 2.3.1. Ethylene Biosynthetic Genes

The expression of the two key genes involved in the biosynthesis of ethylene, *MdACS1*, and *MdACO1* were assessed in ‘Honeycrisp’ fruit subjected to different Extenday and AVG treatment combinations for two different years. 

**Figure 2:**
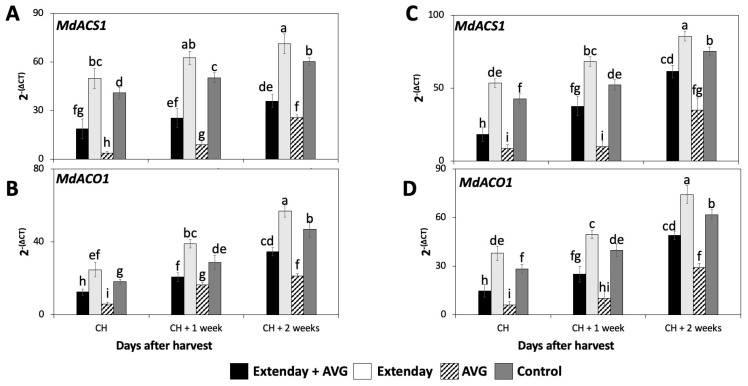
Effects of Extenday and AVG combinations on the relative gene expression levels of ethylene biosynthetic genes of ‘Honeycrisp’ fruit grown in Aspers, PA, in (**A**,**B**) 2021 and (**C**,**D**) 2022. Apples were evaluated at the optimal commercial harvest (CH), 1 week after CH (CH + 1), and 2 weeks after CH (CH + 2). Values are means ± standard error. Different letters indicate significant differences (*p* ≤ 0.05) according to Tukey’s HSD test; 1-aminocyclopropane-carboxylase synthase (ACS), 1-aminocyclopropane-carboxylase oxidase (ACO).

The expression of *MdACS1* and *MdACO1* significantly increased throughout the three assayed ripening stages for all the assessed treatments consistently in both years (Figure 2), following a similar pattern observed for the IEC (Figure 1A,D). In general, for each assessed ripening stage, Extenday-only (T2)-treated fruit always displayed the statistically highest transcript accumulation for *MdACS1* and *MdACO1*, followed by the control (T4) fruit, and next by the combined Extenday + AVG (T1) treatment, while AVG-only (T3)-treated fruit displayed the lowest transcript accumulation for the above-mentioned genes. 

#### 2.3.2. Anthocyanin Biosynthetic Genes and Transcription Factors Associated with Its Regulation

The expression profiles of the seven key structural genes involved in anthocyanin biosynthesis, including *MdPAL*, *MdCHS*, *MdCHI*, *MdF3H*, *MdDFR*, *MdLDOX*, and *MdUFGT*, and one key transcription factor, *MdMYB10*, were assessed in ‘Honeycrisp’ fruit submitted to different Extenday and AVG treatment combinations for two different years. 

**Figure 3:**
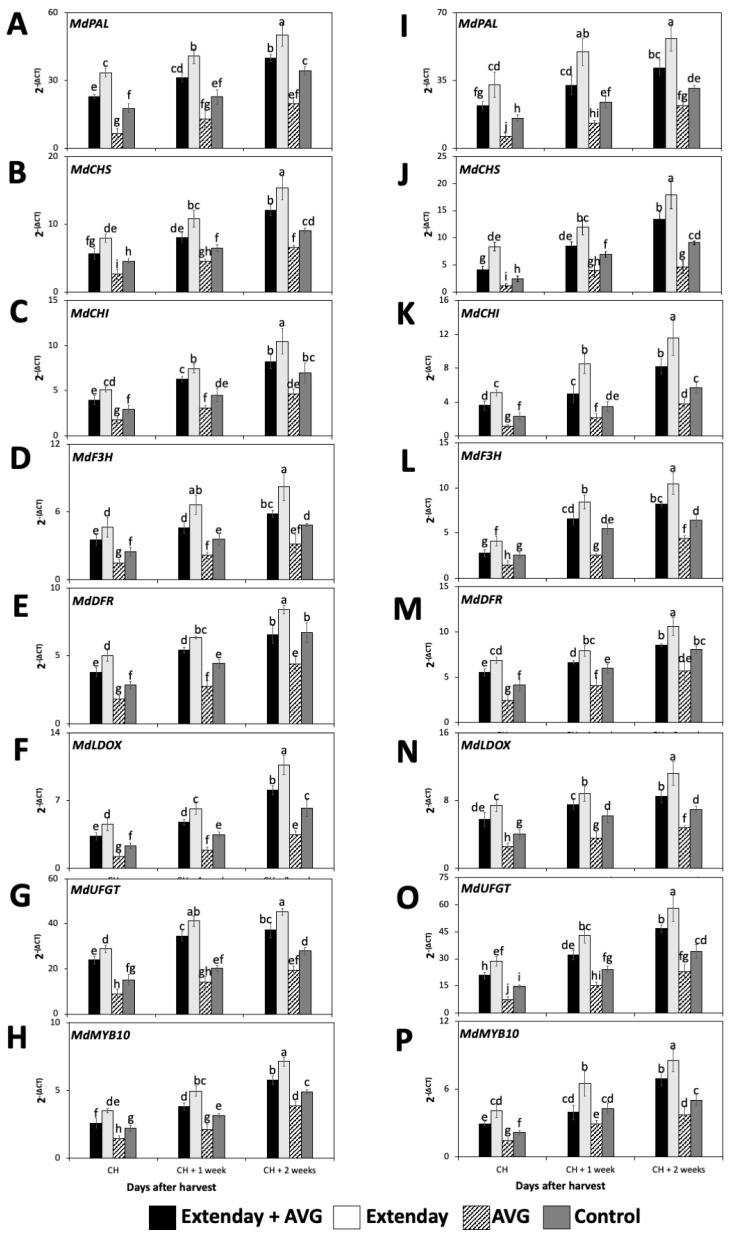
Effects of Extenday and AVG combinations on the relative gene expression levels of anthocyanin structural biosynthetic genes and *MdMYB10* of ‘Honeycrisp’ fruit grown in Aspers, PA, in (**A**–**H**) 2021 and (**I**–**P**) 2022. Apples were evaluated at the optimal commercial harvest (CH), 1 week after CH (CH + 1), and 2 weeks after CH (CH + 2). Values are means ± standard error. Different letters indicate significant differences (*p* ≤ 0.05) according to Tukey’s HSD test. Phenylalanine ammonia-lyase (PAL), chalcone synthase (CHS), chalcone isomerase (CHI), flavanone 3-hydroxylase (F3H), dihydroflavonol 4-reductase (DFR), leucoanthocyanidin dioxygenase (LDOX), UDP glucose-flavonoid 3-O-glucosyltransferase (UFGT).

All the key structural genes and the transcription factors that were evaluated in this study for all treatment combinations displayed a significant increase in the expression profile throughout the three assayed ripening stages, i.e., from CH to CH + 2 (Figure 3). In general, regarding the seven key structural genes, for each assessed ripening stage, the Extenday-only (T2)-treated fruit always displayed the statistically higher transcript accumulation, followed by the combined Extenday + AVG (T1) treatment, and next by the control (T4)-treated fruit, while AVG-only (T3)-treated fruit presented the significantly lowest gene expression values. This was consistent in both years, excluding 2021, where, at CH + 2, fruit submitted to T1 displayed no significant differences with respect to T4 for *MdCHI* and *MdDFR* transcript accumulation (Figure 3C,E). For 2022, at CH and CH + 1, fruit submitted to T1 presented no differences when compared to T4 for *MdF3H* expression profiles (Figure 3L), while, at CH + 2, the transcript accumulation of *MdDFR* was the same for T1 and T4 (Figure 3M).

The transcription factor *MdMYB10* within each evaluation period, and consistently for both years, followed the same expression pattern as the seven assayed structural anthocyanin biosynthesis genes, i.e., the Extenday-only (T2)-treated fruit always displayed the statistically higher transcript accumulation, followed by the combined Extenday + AVG (T1) treatment, and next by the control (T4)-treated fruit, while the AVG-only (T3)-treated fruit presented the significantly lowest *MdMYB10* expression values (Figure 3H,P). This was consistent in both years, excluding 2022, where, at CH + 1, fruit submitted to T1 and T4 displayed no significant differences between them (Figure 3P). 

### 2.4. Effects of Extenday and AVG Treatment Combinations on ‘Honeycrisp’ Total Anthocyanin Concentration

**Figure 4:**
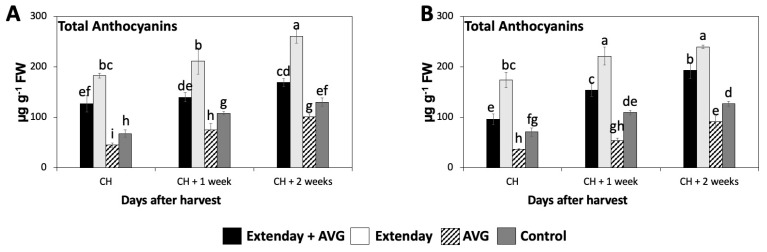
Effects of Extenday and AVG combinations on the total anthocyanin concentration of ‘Honeycrisp’ fruit grown in Aspers, PA, in (**A**) 2021 and (**B**) 2022. Apples were evaluated at the optimal commercial harvest (CH), 1 week after CH (CH + 1), and 2 weeks after CH (CH + 2). Values are means ± standard error. Different letters indicate significant differences (*p* ≤ 0.05) according to Tukey’s HSD test.

The total anthocyanin concentration significantly increased throughout the three assayed ripening stages in this study, resulting in a 1.4- to 2.5-fold increase from CH to CH + 2, considering all the treatment combinations in both years (Figure 4). At all evaluation periods, the Extenday-only (T2)-treated fruit always displayed the significantly highest concentration of total anthocyanins (ranging between 170 and 270 µg g^−1^), followed by the combined Extenday + AVG (T1) treatment (ranging between 100 and 200 µg g^−1^), and next by the control (T4)-treated fruit (ranging between 70 and 130 µg g^−1^), while the AVG-only (T3)-treated fruit presented the significantly lowest values (ranging between 40 and 100 µg g^−1^) consistently in both assayed years (Figure 4).

### 2.5. Relationships among Ethylene Concentration, Red Skin Coloration, Key Ethylene and Anthocyanin Biosynthetic-Related Genes, and Anthocyanin Concentration of ‘Honeycrisp’ Apple Fruit Submitted to Extenday and AVG Treatment Combinations

**Figure 5:**
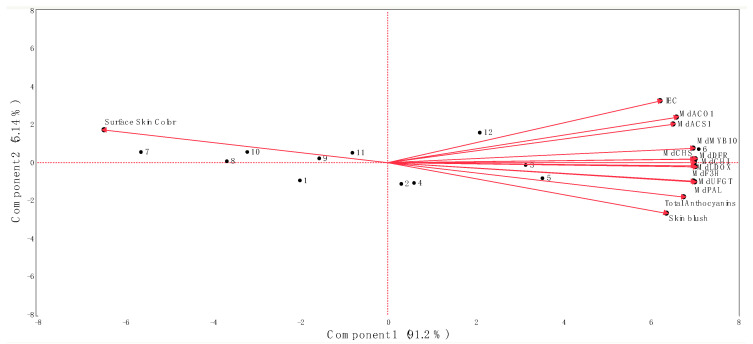
Biplot from principal component analysis of data obtained from the internal ethylene concentration (IEC), red skin coloration, key ethylene and anthocyanin biosynthetic-related genes, and the total anthocyanin concentration of ‘Honeycrisp’ apples submitted to different Extenday and AVG treatments combinations throughout the three ripening stages. Numbers correspond to the different treatments and ripening stages that were assayed (1 (T1_CH), 2 (T1_CH + 1), 3 (T1_CH + 2), 4 (T2_CH), 5 (T2_CH + 1), 6 (T2_CH + 2), 7 (T3_CH), 8 (T3_CH + 1), 9 (T3_CH + 2), 10 (T4_CH), 11 (T4_CH + 1), 12 (T4_CH + 2)). Codes for genes are described in Figure 2 and Figure 3.

The calculation of correlation coefficients was performed (Table 2), and a principal component analysis (PCA) (Figure 5) was conducted considering all the evaluated parameters in this study for ‘Honeycrisp’ apple fruit during 2021 and 2022.

IEC displayed a significant and positive correlation with skin blush (r = 0.63), ethylene biosynthetic-related genes (*MdACS1*, *MdACO1*; r ≥ 0.92), anthocyanin biosynthetic-related genes (*MdPAL*, *MdCHS*, *MdCHI*, *MdF3H*, *MdDFR*, *MdLDOX*, *MdUFGT*; r ≥ 0.80) and transcription factors (*MdMYB10*; r = 0.91), as well as with the total anthocyanin concentration (r = 0.71), while the IEC was negatively correlated with the surface skin hue angle (r = −0.69) (Table 2).

Amongst the color-related parameters, the surface skin hue angle was negatively correlated with skin blush (r = −0.90), ethylene biosynthetic-related genes (r ≤ −0.80), anthocyanin biosynthetic-related genes (r ≤ −0.90), and *MdMYB10* (r = −0.90), as well as with the total anthocyanin concentration (r = −0.90). On the other hand, skin blush displayed significantly positive correlations with ethylene biosynthetic-related genes (r ≥ 0.72), anthocyanin biosynthetic-related genes (r ≥ 0.83), and *MdMYB10* (r = 0.81), as well as with total anthocyanin concentration (r = 0.96). 

Ethylene biosynthetic-related genes presented positive correlations between them (r = 0.98), as well as with anthocyanin biosynthetic-related genes (r ≥ 0.84) and *MdMYB10* (r ≥ 0.90), and with the total anthocyanin concentration (r ≥ 0.81).

All assayed anthocyanin biosynthetic-related genes were positively associated amongst them (r ≥ 0.96), and with the anthocyanin-related transcription factor MdMYB10 (r ≥ 0.95), as well as with the total anthocyanin concentration (r ≥ 0.92). Additionally, the anthocyanin-related transcription factor *MdMYB10* positively correlated with the total anthocyanin concentration (r = 0.90) (Table 2).

The first and second principal components of the PCA explained 91.2% (Component 1) and 5.14% (Component 2) of the total observed variations (96.3%) (Figure 5). The separation of the different treatment combinations assayed in this study along the first principal component of the PCA was determined by the surface skin hue values on the negative side of the axis (associated with treatments Extenday + AVG (T1; at CH), AVG-only (T3; all evaluation periods), and the control (T4; at CH and CH + 1)) and by the IEC, skin blush, all the assessed ethylene and anthocyanin biosynthetic-related genes, and the *MdMYB10* transcription factor, as well as the total anthocyanin concentration on the positive side of the axis (associated with treatments Extenday + AVG (T1; at CH + 1 and CH + 2), Extenday-only (T2; all evaluation periods), and the control (T4; at CH + 2) (Figure 5)).

## 3. Discussion

Anthocyanin accumulation is of major significance throughout the apple fruit ripening process due to its direct influence on red skin coloration, which is associated with a higher apple acceptability and profitability [2,4]. Anthocyanin concentration has been shown to be significantly impacted by light [20,21,22], as well as by ethylene production [23,24,25,26,27], among other factors. In terms of light, the reflective groundcover Extenday has been shown to increase the light that reaches the apple fruit surface [4,40,41] and thus enhances the red skin coloration in apples [3,4,28,39,40,43,44,45,46], but also to increase the ethylene concentration, thus hastening fruit maturity [71,72,73]. In terms of ethylene, the use of AVG has been shown to delay apple ripening rates and to reduce the preharvest fruit drop, a serious problem in ‘Honeycrisp’, which can yield losses up to 50% in the mid-Atlantic by significantly decreasing ethylene production, although it has also been reported to negatively impact apple red skin coloration [4,24,40,54,63,66,67,70]. In our previous study [4], we demonstrated that the combined use of Extenday and AVG treatments could impact the quality and skin color of ‘Honeycrisp’ apples grown under the hot and humid weather of the mid-Atlantic. However, it is still unknown how these treatment combinations impact the expression of key ethylene and anthocyanin biosynthetic genes, as well as the total anthocyanin concentration of ‘Honeycrisp’ apples in the mid-Atlantic US. In this work, the combined use of the reflective groundcover Extenday and the plant growth regulator AVG enhanced the total anthocyanin concentration, as well as the expression of key anthocyanin biosynthetic genes and the transcription factor *MdMYB10*, translating into an increased red skin coloration (>50% blush at CH); moreover, it did not significantly promote the transcript accumulation of key ethylene biosynthetic-related genes, the ethylene concentration, nor the fruit drop to the levels observed in fruit subjected to Extenday-only or control treatments. The later can explain the lack of fruit overripening we observed in our previous study [4] regarding ‘Honeycrisp’ fruit harvested from the lower third of the canopy and subjected to the combined Extenday and AVG treatment (T1). These results were consistent throughout the two consecutive years of study.

The biosynthesis of anthocyanins in red apple cultivars such as ‘Honeycrisp’ has been shown to be regulated at the developmental level, and to primarily take place during fruit ripening [19]. It has been reported that there is a progressive increase in the total anthocyanin accumulation [20], as well as in the expression profiles of the key structural genes (*MdCHS*, *MdF3H*, *MdDFR*, *MdLDOX*, and *MdUFGT*) and transcription factors (*MdMYB10*) involved in anthocyanin biosynthesis [30,34] during fruit ripening. This is consistent with the results for all assayed treatments in this study. Nonetheless, Extenday-only-treated fruit displayed the highest expression levels for anthocyanin biosynthetic-related genes, followed by the Extenday + AVG treatment, while the lowest expression was for AVG-only. These results suggest that the degree of the upregulation of all assayed structural genes for anthocyanin synthesis and for *MdMYB10*, within each examined ripening stage, varied significantly with the use of the different treatment combinations of these horticultural practices. 

The anthocyanin production in apple skin requires the presence of light reaching the surface of the fruit [7,22,74]. In apples, as well as in other fruit, ultraviolet radiation is known to stimulate fruit anthocyanin biosynthesis [21,28,29,31]. Previous research has shown that the use of the reflective groundcover Extenday significantly increases the reflected ultraviolet radiation from the orchard floor towards the tree canopy [3,4,28]. The increased presence of light in the tree canopy can induce anthocyanin biosynthesis and accumulation in apples indirectly by boosting photosynthesis and increasing the supply of assimilates diverted to sink tissues, such as fruit, which consequently provide the substrate for anthocyanin biosynthesis; or directly, via Extenday promoting anthocyanin biosynthesis, as it is known that the majority of the anthocyanin biosynthetic genes and enzymes are significantly upregulated by light [30,32,33]. The latter is in agreement with our results, as the expression levels of the seven assayed structural genes (*MdPAL*, *MdCHS*, *MdCHI*, *MdF3H*, *MdDFR*, *MdLDOX*, and *MdUFGT*) and the key transcription factor *MdMYB10* involved in anthocyanin biosynthesis were markedly and coordinately upregulated in Extenday-only-treated fruit, followed by the combined Extenday + AVG treatment, as compared to the non-Extenday treatments, which displayed the lowest transcript accumulation. Similar results were also observed in ‘Ambrosia’ apples subjected to Extenday treatment during ripening [28]. Consistent with our results, it has been reported that, when comparing apples subjected to higher and lower light intensity treatments, there was a significantly increased expression of anthocyanin biosynthetic-related genes in the former than in the latter conditions [75].

Regulatory genes, such as the transcription factor *MdMYB10*, have been reported to be critical in apple red skin coloration as they control the expression levels of the structural genes involved in the anthocyanin biosynthetic pathway [1,15,16,17,18]. This is supported by the positive correlations obtained amongst *MdMYB10* and all assayed structural anthocyanin biosynthetic-related genes in this work. Furthermore, and consistently with previous studies of apples [19,28,76], there was a positive correlation between the total anthocyanin concentration and the transcript accumulation of the assayed structural genes involved in anthocyanin biosynthesis and *MdMYB10*. Moreover, the expression levels of these key anthocyanin biosynthesis-related genes displayed corresponding increases in the total anthocyanin concentration and in red skin coloration (supported by their positive correlations with skin blush and negative correlations with surface skin hue angle). This is consistent with reports of ‘Ambrosia’ apples subjected to Extenday deployment [28] and with ‘Fortune’ apple fruit in response to increased sunlight exposure [7]. Likewise, in ‘Gala’ apples, anthocyanin accumulation has been shown to be enhanced via the use of Extenday [72]. 

Ethylene production is also known to play a role in regulating the accumulation of anthocyanins [23,24,25,26,30,47,48]. This is supported by the positive correlations obtained in this study between the IEC and ethylene and anthocyanin biosynthetic-related gene expression, *MdMYB10* transcript profiles, the total anthocyanin concentration, as well as with skin blush, and by the negative correlation between the IEC and the surface skin hue angle. The use of reflective groundcovers has been previously shown to increase the IEC, boosting anthocyanin accumulation and thus red skin coloration, but also hastening fruit maturity and therefore fruit overripening [4,33,71,72]. Inversely, the use of AVG, which hinders the activity of ACS activity [54,63], can reduce the IEC, thus delaying fruit maturity as well as the red skin coloration, as has been shown in several apple cultivars, such as ‘Gala’, ‘Jonagold’, ‘Cripps Pink’ [24,40,63,77], ‘McIntosh’ [78], ‘Red Delicious’, and ‘Red Chief’ [70], as well as ‘Honeycrisp’ [4,59]. Consistent with our results, the AVG-only- and the Extenday-only-treated apples presented the significantly lowest and highest transcript accumulation for *MdACS1* and *MdACO1*, as well as for all assayed anthocyanin biosynthetic-related genes and total anthocyanin concentrations at all stages, respectively. These results support the differences obtained in the IEC between these treatments, and also explain the delay of the fruit subjected to the AVG-only treatment to reach the required minimum 50% skin blush (which was only reached at CH + 2). The combined Extenday + AVG treatment displayed gene expression profile values that were positioned in between the above-mentioned treatments, which, for *MdACS1* and *MdACO1*, were only higher than the AVG-only-treated fruit, and the anthocyanin biosynthetic-related genes and total anthocyanin concentration were higher than the control and AVG-only-treated fruit, but lower than the Extenday-only-treated apples. Nevertheless, the combined Extenday + AVG-treated fruit still reached the required 50% blush at CH, therefore emphasizing a potential interactive effect of both horticultural practices. In fact, a synergistic interaction between *MdMYB10* and the ethylene biosynthesis genes, *MdACS1* and *MdACO1*, has been reported, which can then activate the downstream structural genes associated with the anthocyanin biosynthetic pathway, leading to anthocyanin accumulation [24,79,80,81]. Our results suggest that the degree of fine-tuning in the transcript accumulation of ethylene and anthocyanin biosynthesis-related genes in the combined Extenday + AVG treatment allows ‘Honeycrisp’ to comply with the required 50% blush at CH and, at the same time, to advance their maturity throughout ripening, while avoiding overripening, as has been shown to occur with fruit subjected to Extenday-only in our previous study [4].

Ethylene production also has been reported to be crucial in regulating preharvest fruit drop, which is a critical problem affecting the highly susceptible ‘Honeycrisp’ cultivar in many regions, including the mid-Atlantic [59,62,63]. The capacity of AVG to significantly reduce the transcript accumulation of ethylene biosynthetic genes *MdACS1* and *MdACO1*, and therefore of the IEC in ‘Honeycrisp’ fruit, can explain the lower fruit drop percentages that were observed in this study for AVG-only- and for Extenday + AVG-treated apples. Previous studies of ‘Golden Delicious’ apples have shown that fruit drop increases linearly with the rapid increase in ethylene production [65]. In agreement with our results, AVG application (at the same rate and timing as in our work) significantly decreased fruit drop in cultivars such as ‘McIntosh’ [66], ‘Gala’ [40,63], and ‘Honeycrisp’ [59]. The combined Extenday + AVG treatment would consequently warrant that ‘Honeycrisp’ fruit will reach the minimum 50% blush requirement at CH, while reducing fruit drop, and, as previously shown [4], avoiding fruit overripening, hence increasing the fruit value, profitability, and boosting cost/benefit for the grower. 

Concerning the PCA, the positioning of the different treatment combinations/ripening stages along component 1 is supported by the following: the AVG-only-treated ‘Honeycrisp’ fruit exhibiting the significantly lowest *MdACS1* and *MdACO1* expression levels, IEC, red skin coloration, transcript accumulation of anthocyanin biosynthetic-related genes, and total anthocyanin concentration in all assessed ripening stages; the fruit subjected to the combined Extenday + AVG treatment presenting a transitional positioning in terms of ethylene biosynthesis-related gene expression and IEC, but still reaching the required minimum 50% blush at CH, which can be explained by the increased transcript accumulation of anthocyanin biosynthesis-related genes and the total anthocyanin concentration; the control fruit displaying a significant upregulation of *MdACS1* and *MdACO1* and an increased IEC, as compared to the AVG-only and Extenday + AVG treatments, but with a fruit blush that does not reach the minimum 50% requirement at CH, explained by a decreased expression level of anthocyanin biosynthetic-related genes, *MdMYB10*, and total anthocyanin contents when compared to the Extenday treatments; and the fruit subjected to the Extenday-only treatment showing the significantly highest *MdACS1* and *MdACO1* expression levels, IEC, red skin coloration, transcript accumulation of anthocyanin biosynthetic-related genes, and total anthocyanin concentration in all assayed ripening stages. However, and as reported in our previous work [4], Extenday-only-treated fruit also displayed the most advanced fruit maturity, i.e., overripening, followed by the control ‘Honeycrisp’ fruit, with both treatments exhibiting the highest preharvest fruit drop under US mid-Atlantic conditions. The effect of these treatment combinations on other economically important apple cultivars, besides ‘Honeycrisp’ grown under environmental conditions different than the mid-Atlantic US, is currently under investigation. 

## 4. Materials and Methods

### 4.1. Plant Material and Preharvest Orchard Treatments 

This study is a continuation of our previous research [4], and, in the present work, we used the same plant material and preharvest orchard treatments as described therein. In summary, we worked with a 12-year-old ‘Honeycrisp’/‘M9′ apple orchard block trained in a central leader and spaced at 1.5 × 4 m, located in Aspers, PA (39.96° N, 77.28° W). In 2021 and 2022, four treatment combinations, including the reflective groundcover Extenday (Extenday New Zealand, Auckland, New Zealand) and the plant growth regulator AVG (ReTain, Valent Biosciences Corporation, Libertyville, IL, USA), were established. The treatments included T1: Extenday deployed, AVG applied; T2: Extenday deployed, no AVG applied; T3: No Extenday deployed, AVG applied; and T4 (control): No Extenday deployed, no AVG applied. Extenday treatments (T1, T2) were installed contiguous to plots of 50 trees on each side of the row around four weeks before the commercial harvest. Plots with and without Extenday deployment were separated by 30 trees within the tree row, and by 3 rows on each side. AVG treatments (T1, T3) were applied to subplots of 20 trees on Extenday and non-Extenday plots. AVG was applied at a full rate (130 mg L^−1^) four weeks before the commercial harvest. A randomized complete block design with four replications was used.

To collect fruit at the optimal commercial maturity stage, fruit maturity indices were examined each year of the study for ‘Honeycrisp’ fruit during the season [4]. Evaluation periods or harvest dates consisted of three different ripening stages on the tree as follows: optimal commercial harvest (CH), 1 week after CH (CH + 1), and 2 weeks after CH (CH + 2). Each of the four replications per treatment consisted of twenty-five fruit per replication, collected from the lower third of the canopy, as described in our previous work [4]. For each replication, the internal ethylene concentration was assessed in five fruit, which were additionally washed, peeled, and for which the skin tissue was pooled together, frozen and homogenized in liquid nitrogen, and stored at −80 °C for further analysis; the remaining fruit were used to assess the apple skin red coloration.

### 4.2. Fruit Internal Ethylene Concentration and Color Measurements

The fruit internal ethylene concentration (IEC) was measured using 1 mL samples of internal gas from the core cavity of each fruit using a gas chromatograph (GC-2014C, Shimadzu Co., Kyoto, Japan), as described before [4,50,82]. The apple fruit red skin coloration was assessed as previously described [4,50,83].

### 4.3. Fruit Drop Measurements

The fruit drop measurements were conducted as previously described [4]. In general, two weeks before CH, five limbs containing twenty fruit each were tagged (from different sides of the trees and different trees) for each of the replications within each treatment. The fruit drop was assessed weekly by counting the tagged fruit that remained on the limbs from CH through to CH + 2. The fruit drop was estimated as a percentage relative to the initial fruit count per limb.

### 4.4. Real-Time Quantitative RT-PCR Analysis

RNA was isolated from apple fruit skin from each of the four replicates for each treatment and at each harvest date using the cetyltrimethylammonium bromide (CTAB)/NaCl method [84], with some modifications [85,86]. First-strand complementary DNA synthesis, primer design, and quantitative PCR were performed as described before [86]. The sets of primers used for the amplification of the different target genes are listed in Table S1. The analysis of the relative gene expression was performed according to the Comparative Cycle Threshold Method [87]. The transcript values were normalized and calibrated relative to the expression of the reference gene actin (*MdACT*), whose expression did not change across thedifferent treatments and evaluation periods in this study.

### 4.5. Total Anthocyanin Quantification

The total anthocyanins in the apple fruit skin from each of the four replicates for each treatment and at each harvest date were quantified following the method previously described [26]. Absorbance was recorded at 530 nm using a Cary 60 UV–Vis (Agilent Technologies, Palo Alto, CA, USA) spectrophotometer. The concentration of total anthocyanin in the skin samples were determined using a molar extinction coefficient (i.e., 3.43 × 104) for idaein chloride [88], and were expressed as μg g^−1^ fresh weight (i.e., μg g^−1^ FW).

### 4.6. Statistical Analysis

The response variables were modeled using generalized linear mixed models, including treatments and ripening stages as fixed factors, and a block as a random factor to determine the statistical significance of the interactions and main effects (analysis of variance, ANOVA). When the analysis was statistically significant, the separation of means was carried out using Tukey’s HSD test at a significance level of 5%. 

Pearson’s correlation coefficients, using mean-centered data, were calculated for each pairwise combination of the evaluated parameters. PCA, which was applied to reduce the dimensionality of the data, was visualized through a ‘biplot’ graph, thus representing the relationships among the variables (IEC, skin color measurements, gene expression values, anthocyanin contents), as well as the assessed treatments and evaluation periods. The Scree test was used to select the number of principal components that captured most of the variations. The software package JMP (ver 15.2, SAS Institute, Cary, NC, USA) was used for all of the statistical analyses.

## 5. Conclusions

Overall, the treatment combinations of the reflective groundcover Extenday and the plant growth regulator AVG, assayed for two production seasons, significantly altered key ethylene and anthocyanin biosynthesis-related gene expression, the transcript accumulation of the transcription factor *MdMYB10*, as well as the total anthocyanin concentration throughout the ripening of ‘Honeycrisp’ apples grown in the mid-Atlantic US. Correspondingly, these treatments impacted the red skin coloration, IEC, and fruit drop. Extenday-only-treated fruit displayed the highest upregulation of ethylene and anthocyanin biosynthetic-related genes, *MdMYB10*, as well as of the total anthocyanin concentration, thus allowing apples to exceed the required minimum 50% blush at all assayed stages, while also promoting the IEC to a level that has been shown to induce overripening. On the other hand, AVG-only-treated fruit exhibited the significantly lowest expression of key ethylene and anthocyanin biosynthetic structural and regulatory genes, together with the total anthocyanin concentration, preventing apples from reaching the required minimum 50% blush until the last assayed ripening stage (CH + 2), while also decreasing the IEC and fruit drop, and has also been shown to significantly delay ripening. Fruit treated with the combined use of Extenday × AVG fine-tuned the transcript accumulation of ethylene and anthocyanin biosynthetic-related genes, as well as of the total anthocyanin concentration. ‘Honeycrisp’ fruit under Extenday × AVG treatment reached the required 50% blush at CH, reduced fruit drop, and moderately increased the IEC (as compared to only Extenday and control treatments), thus allowing for a delayed harvest while avoiding overripening, as previously shown, hence increasing the ‘Honeycrisp’ crop value and acceptability.

## Figures and Tables

**Table 1:** Effects of Extenday and AVG combinations on the fruit drop of ‘Honeycrisp’ fruit, harvested at three different ripening stages on the tree in Aspers, PA, for two years.

Treatment	Fruit Drop (%)					
	2021			2022		
	CH	CH + 1	CH + 2	CH	CH + 1	CH + 2
T1	6.7 ± 0.4 d	12.0 ± 1.1 c	17.1 ± 0.7 b	4.5 ± 0.3 d	9.9 ± 0.3 c	15.8 ± 0.9 b
T2	10.9 ± 0.7 c	16.3 ± 2.2 b	28.6 ± 2.4 a	5.3± 0.4 d	15.1 ± 1.2 b	25.8 ± 1.9 a
T3	5.7 ± 0.5 d	10.4 ± 1.3 c	16.8 ± 1.8 b	4.7 ± 0.3 d	9.3± 0.6 c	14.9 ± 0.9 b
T4	10.5 ± 1.1 c	17.4 ± 0.7 b	27.1 ± 3.3 a	5.4 ± 0.4 d	15.9 ± 1.2 b	24.8 ± 1.9 a

Apples were harvested at the optimal commercial harvest (CH), 1 week after CH (CH + 1), and 2 weeks after CH (CH + 2). Values are means ± standard error. Different letters indicate significant differences (*p* ≤ 0.05) according to Tukey’s HSD test.

**Table 2:** Pearson correlation coefficients among all assessed features in ‘Honeycrisp’ fruit including the internal ethylene concentration (IEC), red skin coloration, key ethylene and anthocyanin biosynthetic-related genes, and the total anthocyanin concentration.

Feature	IEC	Surface Hue	Skin Blush	*ACS1*	*ACO1*	*PAL*	*CHS*	*CHI*	*F3H*	*DFR*	*LDOX*	*UFGT*	*MYB10*	Total Anthocyanins
IEC	1.00	−0.69 *	0.63 *	0.92 *	0.96 *	0.80 *	0.86 *	0.86 *	0.83 *	0.89 *	0.86 *	0.80*	0.91 *	0.71 *
Surface Hue		1.00	−0.90 *	−0.80 *	−0.81 *	−0.90 *	−0.90 *	−0.90 *	−0.90 *	−0.90 *	−0.90 *	−0.90 *	−0.90 *	−0.90 *
Skin Blush			1.00	0.75 *	0.72 *	0.94 *	0.83 *	0.86 *	0.86 *	0.87 *	0.89 *	0.91 *	0.81 *	0.96 *
*ACS1*				1.00	0.98 *	0.87 *	0.87 *	0.87 *	0.89 *	0.90 *	0.88 *	0.84 *	0.90 *	0.83 *
*ACO1*					1.00	0.87 *	0.89 *	0.89 *	0.90 *	0.92 *	0.89 *	0.85 *	0.93 *	0.81 *
*PAL*						1.00	0.96 *	0.97 *	0.98 *	0.97 *	0.98 *	0.99 *	0.95 *	0.98 *
*CHS*							1.00	0.99 *	0.98 *	0.97 *	0.99 *	0.98 *	0.99 *	0.92 *
*CHI*								1.00	0.98 *	0.97 *	0.99 *	0.99 *	0.99 *	0.93 *
*F3H*									1.00	0.96 *	0.97 *	0.98 *	0.97 *	0.94 *
*DFR*										1.00	0.98 *	0.97 *	0.98 *	0.92 *
*LDOX*											1.00	0.99 *	0.97 *	0.94 *
*UFGT*												1.00	0.96 *	0.96 *
*MYB10*													1.00	0.90 *
Total Anthocyanins														1.00

All correlations shown are significant (* *p* ≤ 0.05).

## Data Availability

The data are available in the article.

## Supplementary Materials

The following supporting information can be downloaded at: https://www.mdpi.com/article/10.3390/plants13081141/s1, Table S1: Primers used in qRT-PCR.

## References

[1] Ban Y., Honda C., Hatsuyama Y., Igarashi M., Bessho H., Moriguchi T. (2007). Isolation and Functional Analysis of a MYB Transcription Factor Gene That Is a Key Regulator for the Development of Red Coloration in Apple Skin. Plant Cell Physiol..

[2] Musacchi S., Serra S. (2018). Apple Fruit Quality: Overview on Pre-Harvest Factors. Sci. Hortic..

[3] Kon T.M., Clavet C.D. (2023). Enhancing Red Fruit Coloration of Apples in the Southeastern US with Reflective Fabrics. Horticulturae.

[4] Miah M.S., Farcuh M. (2024). Combining the Use of Reflective Groundcovers and Aminoethoxyvinylglycine to Assess Effects on Skin Color, Preharvest Drop, and Quality of ‘Honeycrisp’Apples in the Mid-Atlantic US. Horticulturae.

[5] USDA Agricultural Marketing Service Apples Grades and Standards https://www.ams.usda.gov/grades-standards/apple-grades-standards.

[6] Castañeda-Ovando A., de Lourdes Pacheco-Hernández M., Páez-Hernández M.E., Rodríguez J.A., Galán-Vidal C.A. (2009). Chemical Studies of Anthocyanins: A Review. Food Chem..

[7] Feng F., Li M., Ma F., Cheng L. (2013). Phenylpropanoid Metabolites and Expression of Key Genes Involved in Anthocyanin Biosynthesis in the Shaded Peel of Apple Fruit in Response to Sun Exposure. Plant Physiol. Biochem..

[8] Rupasinghe H.P. (2020). Special Issue “Flavonoids and Their Disease Prevention and Treatment Potential”: Recent Advances and Future Perspectives. Molecules.

[9] Li P., Cheng L. (2008). The Shaded Side of Apple Fruit Becomes More Sensitive to Photoinhibition with Fruit Development. Physiol. Plant..

[10] Boyer J., Liu R.H. (2004). Apple Phytochemicals and Their Health Benefits. Nutr. J..

[11] Hyson D.A. (2011). A Comprehensive Review of Apples and Apple Components and Their Relationship to Human Health. Adv. Nutr..

[12] Łata B., Trampczynska A., Paczesna J. (2009). Cultivar Variation in Apple Peel and Whole Fruit Phenolic Composition. Sci. Hortic..

[13] Kunradi Vieira F.G., da Silva Campelo Borges G., Copetti C., Valdemiro Gonzaga L., da Costa Nunes E., Fett R. (2009). Activity and Contents of Polyphenolic Antioxidants in the Whole Fruit, Flesh and Peel of Three Apple Cultivars. Arch. Latinoam. Nutr..

[14] Xie R., Zheng L., He S., Zheng Y., Yi S., Deng L. (2011). Anthocyanin Biosynthesis in Fruit Tree Crops: Genes and Their Regulation. Afr. J. Biotechnol..

[15] Espley R.V., Brendolise C., Chagne D., Kutty-Amma S., Green S., Volz R., Putterill J., Schouten H.J., Gardiner S.E., Hellens R.P. (2009). Multiple Repeats of a Promoter Segment Causes Transcription Factor Autoregulation in Red Apples. Plant Cell.

[16] Telias A., Lin-Wang K., Stevenson D.E., Cooney J.M., Hellens R.P., Allan A.C., Hoover E.E., Bradeen J.M. (2011). Apple Skin Patterning Is Associated with Differential Expression of MYB10. BMC Plant Biol..

[17] Ryu J.-A., Duan S., Gil C.S., Jeong H.Y., Lee C., Kang I.-K., Eom S.H. (2022). Combined UV-B and Methyl Jasmonate Treatments Enhance Postharvest Pigmentation of “Fuji” Apples. Postharvest Biol. Technol..

[18] Espley R.V., Hellens R.P., Putterill J., Stevenson D.E., Kutty-Amma S., Allan A.C. (2007). Red Colouration in Apple Fruit Is Due to the Activity of the MYB Transcription Factor, MdMYB10. Plant J..

[19] Honda C., Kotoda N., Wada M., Kondo S., Kobayashi S., Soejima J., Zhang Z., Tsuda T., Moriguchi T. (2002). Anthocyanin Biosynthetic Genes Are Coordinately Expressed during Red Coloration in Apple Skin. Plant Physiol. Biochem..

[20] Ubi B.E., Honda C., Bessho H., Kondo S., Wada M., Kobayashi S., Moriguchi T. (2006). Expression Analysis of Anthocyanin Biosynthetic Genes in Apple Skin: Effect of UV-B and Temperature. Plant Sci..

[21] Honda C., Moriya S. (2018). Anthocyanin Biosynthesis in Apple Fruit. Hortic. J..

[22] Lancaster J.E. (1992). Regulation of Skin Color in Apples. CRC. Crit. Rev. Plant Sci..

[23] Farcuh M., Tajima H., Lerno L.A., Blumwald E. (2022). Changes in Ethylene and Sugar Metabolism Regulate Flavonoid Composition in Climacteric and Non-Climacteric Plums during Postharvest Storage. Food Chem. Mol. Sci..

[24] Wang Z., Dilley D.R. (2001). Aminoethoxyvinylglycine, Combined with Ethephon, Can Enhance Red Color Development without over-Ripening Apples. HortScience.

[25] Blankenship S.M., Unrath C.R. (1988). PAL and Ethylene Content during Maturation of Red and Golden Delicious Apples. Phytochemistry.

[26] Whale S.K., Singh Z. (2007). Endogenous Ethylene and Color Development in the Skin of ‘Pink Lady’Apple. J. Am. Soc. Hortic. Sci..

[27] Whale S.K., Singh Z., Behboudian M.H., Janes J., Dhaliwal S.S. (2008). Fruit Quality in ‘Cripp’s Pink’Apple, Especially Colour, as Affected by Preharvest Sprays of Aminoethoxyvinylglycine and Ethephon. Sci. Hortic..

[28] Toivonen P., Stoochnoff J., Usher K., Lu C., Wiersma P., Zhou C. (2019). Biochemical and Gene Expression Involved in Red Blush Color Development in ‘Ambrosia’ Apple. J. Am. Soc. Hortic. Sci..

[29] Charles M.T., Arul J. (2007). UV Treatment of Fresh Fruits and Vegetables for Improved Quality: A Status Report. Stewart Postharvest Rev..

[30] Chen Z., Yu L., Liu W., Zhang J., Wang N., Chen X. (2021). Research Progress of Fruit Color Development in Apple (*Malus domestica borkh*). Plant Physiol. Biochem..

[31] Dong Y., Mitra D., Kootstra A., Lister C., Lancaster J. (1995). Postharvest Stimulation of Skin Color in Royal Gala Apple. J. Am. Soc. Hortic. Sci..

[32] Vimolmangkang S., Zheng D., Han Y., Khan M.A., Soria-Guerra R.E., Korban S.S. (2014). Transcriptome Analysis of the Exocarp of Apple Fruit Identifies Light-Induced Genes Involved in Red Color Pigmentation. Gene.

[33] Ju Z., Duan Y., Ju Z. (1999). Effects of Covering the Orchard Floor with Reflecting Films on Pigment Accumulation and Fruit Coloration InFuji’apples. Sci. Hortic..

[34] Kondo S., Hiraoka K., Kobayashi S., Honda C., Terahara N. (2002). Changes in the Expression of Anthocyanin Biosynthetic Genes during Apple Development. J. Am. Soc. Hortic. Sci..

[35] Takos A.M., Jaffé F.W., Jacob S.R., Bogs J., Robinson S.P., Walker A.R. (2006). Light-Induced Expression of a MYB Gene Regulates Anthocyanin Biosynthesis in Red Apples. Plant Physiol..

[36] Allan A.C., Hellens R.P., Laing W.A. (2008). MYB Transcription Factors That Colour Our Fruit. Trends Plant Sci..

[37] Xu Y., Feng S., Jiao Q., Liu C., Zhang W., Chen W., Chen X. (2012). Comparison of MdMYB1 Sequences and Expression of Anthocyanin Biosynthetic and Regulatory Genes between Malus Domestica Borkh. Cultivar ‘Ralls’ and Its Blushed Sport. Euphytica.

[38] Funke K., Blanke M. (2021). Spatial and Temporal Enhancement of Colour Development in Apples Subjected to Reflective Material in the Southern Hemisphere. Horticulturae.

[39] Mupambi G., Valverdi N.A., Camargo-Alvarez H., Reid M., Kalcsits L., Schmidt T., Castillo F., Toye J. (2021). Reflective Groundcover Improves Fruit Skin Color in ‘Honeycrisp’Apples Grown under Protective Netting. Horttechnology.

[40] Layne D.R., Jiang Z., Rushing J.W. (2002). The Influence of Reflective Film and ReTain on Red Skin Coloration and Maturity OfGala’Apples. Horttechnology.

[41] Privé J.-P., Russell L., LeBlanc A. (2011). Impact of Reflective Groundcover on Growth, Flowering, Yield and Fruit Quality in Gala Apples in New Brunswick. Can. J. Plant Sci..

[42] Toye J. (1995). Reflective Mulches—New Zealand Leads the Way. Orchard..

[43] Iglesias I., Alegre S. (2009). The Effects of Reflective Film on Fruit Color, Quality, Canopy Light Distribution, and Profitability of “Mondial Gala” Apples. Horttechnology.

[44] Robinson T.L., Gonzalez L. (2023). Effect of Different Reflective Ground Covers on Light Reflection and on the Coloring of Apples at Harvest. Proc. Acta Hortic..

[45] Privé J.P., Russell L., Leblanc A. (2008). Use of Extenday Reflective Groundcover in Production of ‘Gala’ Apples (*Malus domestica*) in New Brunswick, Canada: 1. Impact on Canopy Microclimate and Leaf Gas Exchange. N. Z. J. Crop Hortic. Sci..

[46] Miller S.S., Greene G.M. (2003). The Use of Reflective Film and Ethephon to Improve Red Skin Color of Apples in the Mid-Atlantic Region of the United States. Horttechnology.

[47] Shafiq M., Singh Z., Khan A.S. (2014). Pre-Harvest Ethephon Application and Training Systems Affect Colour Development, Accumulation of Flavonoids and Fruit Quality of ‘Cripps Pink’ apple. Aust. J. Crop Sci..

[48] Faragher J.D., Brohier R.L. (1984). Anthocyanin Accumulation in Apple Skin during Ripening: Regulation by Ethylene and Phenylalanine Ammonia-Lyase. Sci. Hortic..

[49] Burg S.P., Burg E.A. (1965). Ethylene Action and the Ripening of Fruits: Ethylene Influences the Growth and Development of Plants and Is the Hormone Which Initiates Fruit Ripening. Science.

[50] Miah M.S., Hinson C., Farcuh M. (2023). Assessing Fruit Maturity and Quality of ‘Buckeye Gala’ Grown on a Diverse Panel of Apple (*Malus domestica borkh*) Rootstocks in Western Maryland. Agronomy.

[51] Farcuh M., Copes B., Le-Navenec G., Marroquin J., Cantu D., Bradford K.J., Guinard J.-X., Van Deynze A. (2020). Sensory, Physicochemical and Volatile Compound Analysis of Short and Long Shelf-Life Melon (*Cucumis melo* L.) Genotypes at Harvest and after Postharvest Storage. Food Chem. X.

[52] Farcuh M., Li B., Rivero R.M., Shlizerman L., Sadka A., Blumwald E. (2017). Sugar Metabolism Reprogramming in a Non-Climacteric Bud Mutant of a Climacteric Plum Fruit during Development on the Tree. J. Exp. Bot..

[53] Farcuh M., Toubiana D., Sade N., Rivero R.M., Doron-Faigenboim A., Nambara E., Sadka A., Blumwald E. (2019). Hormone Balance in a Climacteric Plum Fruit and Its Non-Climacteric Bud Mutant during Ripening. Plant Sci..

[54] Arseneault M.H., Cline J.A. (2016). A Review of Apple Preharvest Fruit Drop and Practices for Horticultural Management. Sci. Hortic..

[55] Brumos J. (2021). Gene Regulation in Climacteric Fruit Ripening. Curr. Opin. Plant Biol..

[56] Costa F., Stella S., Van de Weg W.E., Guerra W., Cecchinel M., Dallavia J., Koller B., Sansavini S. (2005). Role of the Genes Md-ACO1 and Md-ACS1 in Ethylene Production and Shelf Life of Apple (*Malus domestica borkh*). Euphytica.

[57] Yang S.F., Hoffman N.E. (1984). Ethylene Biosynthesis and Its Regulation in Higher Plants. Annu. Rev. Plant Physiol..

[58] Cherian S., Figueroa C.R., Nair H. (2014). ‘Movers and Shakers’ in the Regulation of Fruit Ripening: A Cross-Dissection of Climacteric versus Non-Climacteric Fruit. J. Exp. Bot..

[59] Arseneault M.H., Cline J.A. (2018). AVG, NAA, Boron, and Magnesium Influence Preharvest Fruit Drop and Fruit Quality of ‘Honeycrisp’ Apples. Can. J. Plant Sci..

[60] Chu C.L. (1988). Internal Ethylene Concentration of ‘McIntosh’, ‘Northern Spy’, ‘Empire’, ‘Mutsu’, and ‘Idared’ Apples during the Harvest Season. J. Am. Soc. Hortic. Sci..

[61] Gussman C.D., Goffreda J.C., Gianfagna T.J. (1993). Ethylene Production and Fruit-Softening Rates in Several Apple Fruit Ripening Variants. HortScience.

[62] Irish-Brown A., Schwallier P., Shane B., Tritten B. (2011). Why Does Apple Fruit Drop Prematurely?.

[63] Liu J., Islam M.T., Sherif S.M. (2022). Effects of Aminoethoxyvinylglycine (AVG) and 1-Methylcyclopropene (1-MCP) on the Pre-Harvest Drop Rate, Fruit Quality, and Stem-End Splitting in ‘Gala’ Apples. Horticulturae.

[64] Schupp J.R., Greene D.W. (2004). Effect of Aminoethoxyvinylglycine (AVG) on Preharvest Drop, Fruit Quality, and Maturation of ‘McIntosh’ Apples. I. Concentration and Timing of Dilute Applications of AVG. HortScience.

[65] Li J., Zhu H., Yuan R. (2010). Profiling the Expression of Genes Related to Ethylene Biosynthesis, Ethylene Perception, and Cell Wall Degradation during Fruit Abscission and Fruit Ripening in Apple. J. Am. Soc. Hortic. Sci..

[66] Greene D.W., Schupp J.R. (2004). Effect of Aminoethoxyvinylglycine (AVG) on Preharvest Drop, Fruit Quality, and Maturation of McIntosh’ Apples. II. Effect of Timing and Concentration Relationships and Spray Volume. HortScience.

[67] Greene D.W. (2005). Time of Aminoethoxyvinylglycine Application Influences Preharvest Drop and Fruit Quality of ‘McIntosh’ apples. HortScience.

[68] Byers R.E. (1997). Effects of Aminoethoxyvinylglycine (AVG) on Preharvest Fruit Drop, Maturity, and Cracking of Several Apple Cultivars. J. Tree Fruit. Prod..

[69] Yuan R., Li J. (2008). Effect of Sprayable 1-MCP, AVG, and NAA on Ethylene Biosynthesis, Preharvest Fruit Drop, Fruit Maturity, and Quality of ‘Delicious’ Apples. HortScience.

[70] Boyacı S. (2022). Effect of Aminoethoxyvinylglycine (AVG) Applications on Pre-Harvest Drop and Fruit Quality of ‘Red Delicious, Red Chief’ Apple Cultivar. Erwerbs-Obstbau.

[71] Layne D.R., Jiang Z., Rushing J.W. (2001). Tree Fruit Reflective Film Improves Red Skin Coloration and Advances Maturity in Peach. Horttechnology.

[72] Overbeck V., Schmitz-Eiberger M.A., Blanke M.M. (2013). Reflective Mulch Enhances Ripening and Health Compounds in Apple Fruit. J. Sci. Food Agric..

[73] Crisosto C.H., Mitchell F.G., Ju Z. (1999). Susceptibility to Chilling Injury of Peach, Nectarine, and Plum Cultivars Grown in California. HortScience.

[74] Saure M.C. (1990). External Control of Anthocyanin Formation in Apple. Sci. Hortic..

[75] An J.-P., Liu Y.-J., Zhang X.-W., Bi S.-Q., Wang X.-F., You C.-X., Hao Y.-J. (2020). Dynamic Regulation of Anthocyanin Biosynthesis at Different Light Intensities by the BT2-TCP46-MYB1 Module in Apple. J. Exp. Bot..

[76] Lister C.E., Lancaster J.E., Walker J.R.L. (1996). Developmental Changes in Enzymes of Flavonoid Biosynthesis in the Skins of Red and Green Apple Cultivars. J. Sci. Food Agric..

[77] Phan-Thien K.Y., Wargo J.M., Mitchell L.W., Collett M.G., Rath A.C. (2004). Delay in Ripening of Gala and Pink Lady Apples in Commercial Orchards Following Pre-Harvest Applications of Aminoethoxyvinylglycine. Aust. J. Exp. Agric..

[78] Stover E., Fargione M.J., Watkins C.B., Iungerman K.A. (2003). Harvest Management of ‘Marshall McIntosh’ Apples: Effects of AVG, NAA, Ethephon, and Summer Pruning on Preharvest Drop and Fruit Quality. HortScience.

[79] Yu J., Qiu K., Sun W., Yang T., Wu T., Song T., Zhang J., Yao Y., Tian J. (2022). A Long Noncoding RNA Functions in High-Light-Induced Anthocyanin Accumulation in Apple by Activating Ethylene Synthesis. Plant Physiol..

[80] An J.-P., Wang X.-F., Li Y.-Y., Song L.-Q., Zhao L.-L., You C.-X., Hao Y.-J. (2018). EIN3-LIKE1, MYB1, and ETHYLENE RESPONSE FACTOR3 Act in a Regulatory Loop That Synergistically Modulates Ethylene Biosynthesis and Anthocyanin Accumulation. Plant Physiol..

[81] Awad M.A., De Jager A. (2002). Formation of Flavonoids, Especially Anthocyanin and Chlorogenic Acid in ‘Jonagold’ Apple Skin: Influences of Growth Regulators and Fruit Maturity. Sci. Hortic..

[82] Farcuh M., Hopfer H. (2023). Aroma Volatiles as Predictors of Chilling Injury Development during Peach (*Prunus persica* (L) Batsch) Cold Storage and Subsequent Shelf-Life. Postharvestig. Biol. Technol..

[83] Infante R., Farcuh M., Meneses C. (2008). Monitoring the Sensorial Quality and Aroma through an Electronic Nose in Peaches during Cold Storage. J. Sci. Food Agric..

[84] Chang S., Puryear J., Cairney J. (1993). A Simple and Efficient Method for Isolating RNA from Pine Trees. Plant Mol. Biol. Report..

[85] Farcuh M., Rivero R.M., Sadka A., Blumwald E. (2018). Ethylene Regulation of Sugar Metabolism in Climacteric and Non-Climacteric Plums. Postharvestig. Biol. Technol..

[86] Kim H., Farcuh M., Cohen Y., Crisosto C., Sadka A., Blumwald E. (2015). Non-Climacteric Ripening and Sorbitol Homeostasis in Plum Fruits. Plant Sci..

[87] Livak K.J., Schmittgen T.D. (2001). Analysis of Relative Gene Expression Data Using Real-Time Quantitative PCR and the 2^−ΔΔCT^ Method. Methods.

[88] Siegelman H.W., Hendricks S.B. (1958). Photocontrol of Anthocyanin Synthesis in Apple Skin. Plant Physiol..

