# Supplementary material for: The Expression of Key Ethylene and Anthocyanin Biosynthetic Genes of ‘Honeycrisp’ Apples Subjected to the Combined Use of Reflective Groundcovers and Aminoethoxyvinylglycine in the Mid-Atlantic US
Source: Plants (Basel). 2024 Apr 19;13(8):1141. doi: 10.3390/plants13081141 (PMC11054659; doi:10.3390/plants13081141)
Supplement: Supplementary file 1 [file plants-13-01141-s001.zip › plants-2957148-supplementary.pdf]

**Supplementary Table S1.** Primers used in qRT-PCR

| Gene name | Description | Primer orientation | Primer sequence (5' to 3') |
|---|---|---|---|
| *MdPAL* | Phenylalanine ammonia-lyase | Forward | GTGCTGTGGAGTCCCCGCTT |
| | | Reverse | GGTGA GGCTCTCTCCGCCAAGT |
| *MdCHS* | Chalcone synthase | Forward | GGAGACAACTGGAGAAGGACTGGAA |
| | | Reverse | CGACATTGATACTGGTGTCTTCA |
| *MdCHI* | Chalcone isomerase | Forward | GGGATAACCTCGCGGCCAAA |
| | | Reverse | GCATCCATGCCGGAAGCTACAA |
| *MdF3H* | Flavanone 3-hydroxylase | Forward | TGGAAGCTTGTGAGGACTGGGGT |
| | | Reverse | CTCCTCCGATGGCAAATCAAAGA |
| *MdDFR* | Dihydroflavonol 4-reductase | Forward | GATAGGGTTTGAGTTCAAGTA |
| | | Reverse | TCTCCTCAGCAGCCTCAGTTTTCT |
| *MdLDOX* | Leucoanthocyanidin dioxygenase | Forward | CCAAGTGAAGCGGGTTGTGCT |
| | | Reverse | CAAAGCAGGCGGACAGGAGTAGC |
| *MdUFGT* | UDP glucose-flavonoid 3- o -glucosyl transferase | Forward | CCACCGCCCTTCCAAACACTCT |
| | | Reverse | CACCCTTATGTTACGCGGCATGT |
| *MdMYB10* | Transcription factor | Forward | TGCCTGGACTCGAGAGGAAGACA |
| | | Reverse | CCTGTTTCCCAAAAGCCTGTGAA |
| *MdACS1* | 1-aminocyclopropane-carboxylase (ACC) synthase | Forward | CTCCTCCTTTCCTTCGTTGA |
| | | Reverse | ACCATGTCGTCGTTGGAGTAG |
| *MdACO1* | ACC oxidase | Forward | ATCAATGATGCTTGTGAGAACTG |
| | | Reverse | GGTCTTCTTGTAGTGATCCTTGG |
| *MdACT* | Actin | Forward | TGACCGAATGAGCAAGGAAATTACT |
| | | Reverse | TACTCAGCTTTGGCAATCCACATC |
